# Correction: Domestication and lowland adaptation of coastal preceramic maize from Paredones, Peru

**DOI:** 10.7554/eLife.91314

**Published:** 2023-07-25

**Authors:** Miguel Vallebueno-Estrada, Guillermo G Hernández-Robles, Eduardo González-Orozco, Ivan Lopez-Valdivia, Teresa Rosales Tham, Víctor Vásquez Sánchez, Kelly Swarts, Tom D Dillehay, Jean-Philippe Vielle-Calzada, Rafael Montiel

**Keywords:** Maize

Vallebueno-Estrada M, Hernández-Robles GG, González-Orozco E, Lopez-Valdivia I, Rosales Tham T, Vásquez Sánchez V, Swarts K, Dillehay TD, Vielle-Calzada J-P, Montiel R. 2023. Domestication and lowland adaptation of coastal preceramic maize from Paredones, Peru. *eLife* **12**:e83149. doi: 10.7554/eLife.83149.

Published 18 April 2023

We noticed that in Figure 1C, one of the samples (Par-N1) was 25% bigger than its actual scale. Although this detail does not impact any of the claims drawn in the paper, we would like to correct it to ensure accurate information about the phenotype of the samples for future studies.

The corrected Figure 1 is shown here:

**Figure 1:**
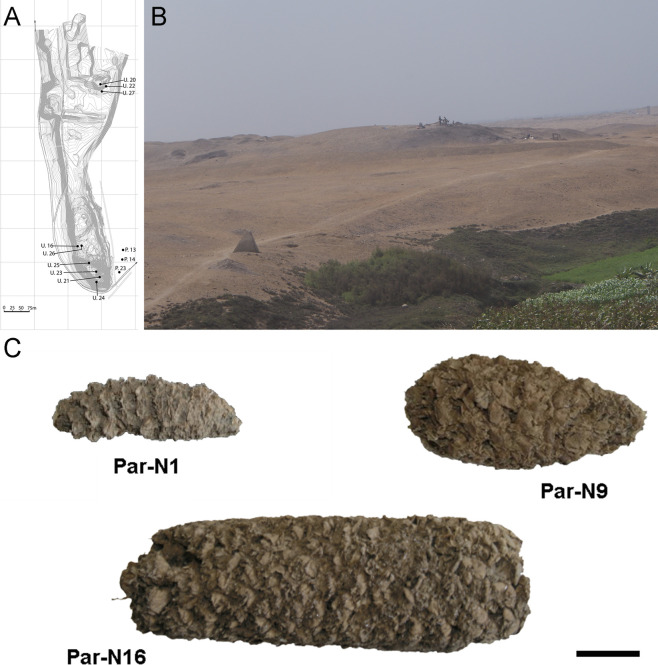


The originally published Figure 1 is shown for reference:

**Figure 2:**
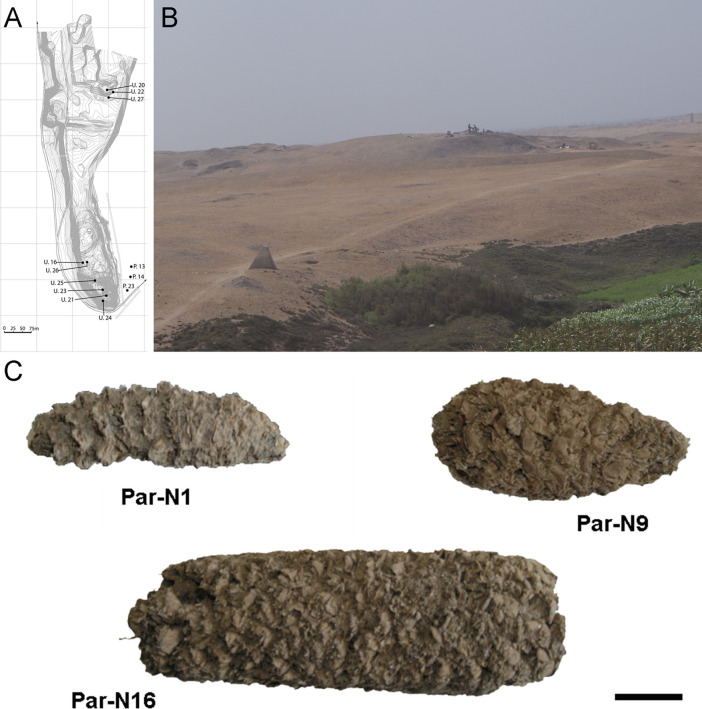


The article has been corrected accordingly.

